# Cocaine disrupts hidden states in the brain

**DOI:** 10.7554/eLife.111296

**Published:** 2026-04-13

**Authors:** Margo Le, Ronald Keiflin

**Affiliations:** 1 Department of Psychological and Brain Sciences, University of California, Santa Barbara Santa Barbara United States

**Keywords:** substance use disorder, cocaine, orbitofrontal cortex, single-unit recording, Rat

## Abstract

Cocaine use disrupts the encoding of abstract states in the orbitofrontal cortex.

**Related research article** Zong W, Mueller LE, Zhang Z, Zhou J, Schoenbaum G. 2026. Prior cocaine use disrupts identification of hidden states by single units and neural ensembles in orbitofrontal cortex. *eLife* **15**:RP109883. doi: 10.7554/eLife.109883.

Associative learning refers to the ability of animals – including humans – to learn relationships between cues and outcomes. However, these relationships often depend on information that is not directly observable. The same cue may predict different outcomes depending on context, while different cues can sometimes predict the same outcome. To guide behavior, the brain must therefore infer the current “hidden state” – an internal representation that captures the structure of the task beyond immediate sensory input (Langdon et al., 2019).

Recent work has suggested that the representation of the hidden state is a core function of the orbitofrontal cortex (OFC), a brain region involved in flexible, adaptive decision making. (Bradfield et al., 2015; Schiereck et al., 2026; Schuck et al., 2016; Zhou et al., 2021). Damage to the OFC is linked to inflexible or maladaptive behavior, and similar impairments have been observed in individuals with substance use disorders, including addiction to cocaine or amphetamines. However, it has so far remained unclear how hidden state representations in the OFC are altered following drug exposure.

Now, in eLife, Wenhui Zong (National Institute on Drug Abuse; NIDA), Lauren Mueller (NIDA), Zhewei Zhang (NIDA), Jingfeng Zhou (Beijing Normal University) and Geoffrey Schoenbaum (NIDA) report experiments in rats showing how this function is altered by prior drug exposure (Zong et al., 2026). The researchers recorded the activity from thousands of OFC neurons at a single-neuron resolution in two groups of rats: a control group with no previous drug exposure, and a group that had previously self-administered cocaine. Neural activity was measured as both groups performed an odor-guided task in which some odors predicted a reward, whereas others did not. Rats in both groups readily learned to distinguish between these cues.

Importantly, the task was more complex than a simple cue-reward association. Odors were presented in two alternating sequences of four. The first and last odors were unique to each sequence, whereas the middle two were the same in both sequences. An intuitive way to think about this is as two bus routes. Each odor (that is, each trial) is analogous to a stop along the route: some stops are unique to a specific route, while others are shared between routes (Figure 1).

**Figure 1.**
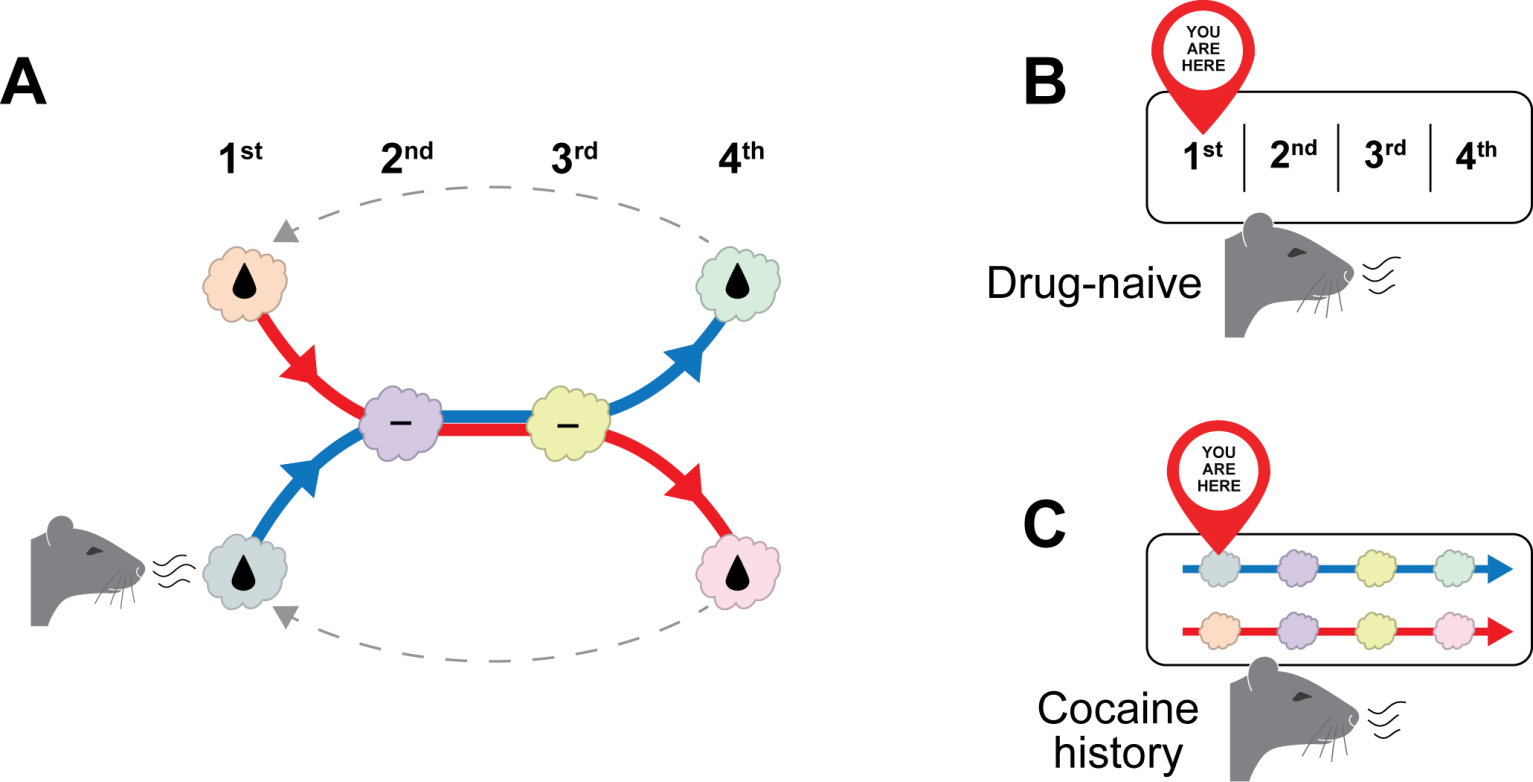
Cocaine self-administration disrupts hidden state representations in the orbitofrontal cortex of rats. (**A**) Zong et al. studied two groups of rats: drug-naïve rats and rats with a history of cocaine self-administration. Both groups were trained in an odor-guided task in which some odors predicted reward (indicated by a droplet symbol) and others did not. Odors were presented in two alternating sequences. In a bus-route analogy, each trial corresponds to a stop along one of two routes: some stops are unique to a given route, while others are shared between routes. (**B, C**). Decoding of orbitofrontal cortex (OFC) population activity. In drug-naïve rats (**B**), OFC activity tracked the animal’s abstract position within the task, generalizing across trial (“stop”) and sequence (“route”) identity, consistent with a representation of the underlying hidden state. In rats with a history of cocaine self-administration (**C**), OFC activity was more tightly linked to specific trials and sequences, reflecting a failure to capture the abstract structure of the task and a breakdown in hidden state representation.

Zong et al. then analyzed how OFC neuronal ensembles encode the animal’s state during the task. At one extreme, the neurons may treat each trial as an independent event, encoding only the identity of each odor (or bus stop), and failing to capture the structure of the task. Alternatively, the neurons could encode trials in a sequence-specific manner, distinguishing between identical odors (or bus stops) based on the current sequence (or bus route). Finally, the neurons might represent a more abstract concept: the trial number, regardless of the specific sequence (“first stop”, “second stop”, etc.). In this case, the representation is informed by the immediate sensory input but not strictly determined by it, reflecting knowledge of the underlying hidden state.

Using sophisticated analyses of neural population activity, Zong et al. found that the rats who had not been exposed to drugs favored the more abstract strategy, with OFC activity reflecting the animal’s hidden state rather than the specific features of individual trials or sequences. In contrast, rats with a history of cocaine self-administration showed a different pattern, with their OFC activity more tightly linked to specific trials and sequences, emphasizing sensory differences rather than conceptual commonalities across situations (Figure 1). This suggests a reduced ability to generalize across situations that occupy the same conceptual position in the task. Notably, this impairment was observed several weeks after rats’ last exposure to cocaine.

What does this mean for behavior? In the study of Zong et al., behavioral differences between groups were minimal, which is important because it indicates that the observed neural differences were not simply due to overt differences in performance. However, cognitive deficits following cocaine use are well documented, and impaired hidden-state representation may be one of the root causes of these deficits (Lucantonio et al., 2014; Schoenbaum and Setlow, 2005; Wied et al., 2013). This could have substantial implications for addiction. One possible consequence is a failure to generalize learning across contexts: knowledge gained in one situation may not transfer to another, even when the two situations are functionally equivalent and should be represented as the same hidden state. Such failures of generalization could contribute to persistent maladaptive behavior and complicate recovery.

Overall, these findings identify hidden-state representation as a fundamental function of the OFC and demonstrate how cocaine disrupts this process. Interventions – behavioral or pharmacological – aimed at restoring this function might be promising strategies for treating addiction (Lucantonio et al., 2014; Panayi et al., 2024).
